# Introduction to Special Issue on “Fluorescence-Based Sensing Technologies”

**DOI:** 10.3390/bios5040616

**Published:** 2015-10-10

**Authors:** A. Sheila Holmes-Smith

**Affiliations:** Department of Engineering, School of Engineering and Built Environment, Glasgow Caledonian University, Cowcaddens Road, Glasgow G4 0BA, UK; E-Mail: a.s.smith@gcu.ac.uk; Tel.: +44-141-331-3552

The application of fluorescence-based technologies to sensing applications in biosciences and related industries is growing. This is mainly due to the high sensitivity of fluorescence and the selectivity that functionalised sensing polymers can offer. The articles published in this Special Issue demonstrate the diverse application of fluorescence to sensing in a broad biological arena.

In the medical application field, a sensor for the detection of the antibiotic drug, Rapamycin, is presented [1]. The authors present a novel detection method using the fluorescence quench-release principle pioneered by the research group and applied to the detection of a range of antigens. They have termed this methodology “Q-body”. In this paper they describe the construction of the Rapamycin sensor and present results to show the ability of the sensor to detect of Rapamycin at therapeutic concentrations.

A review of total internal reflection fluorescence (TIRF) microscopy is given by Fang [2]. In this review the author gives an up-to-date summary of the application of this fluorescence-based method to receptor pharmacology. Included in the paper is a good summary of the theory of evanescent wave sensing and a description of instrument configurations employing this technique. A review of the application of this methodology to cell biology and pharmacological profiling are presented.

Suzuki and Yokoyama [3] have reviewed functionalised molecular probes for the detection of biological substances. The review concentrates on metal ion selective fluorescent probes which have been developed for the physiological important metal ions: calcium, magnesium and zinc. In addition fluorescent molecular probes for the detection of proteins are also presented.

Away from the medical applications of biosensors is the extremely important area of sensing within the food industry where there is a need for the development of sensors for foodstuffs. This review presents how time-resolved fluorescence measurements can be applied to monitoring bioactive compounds in plant-based food stuffs [4]. The review describes time-resolved fluorescence techniques which are applicable to many biosensing applications and as such provides a useful reference. This particular review focuses on the changes in the fluorescence properties of bioactive compounds in various vegetables with different storage conditions and cooking.

Andrus *et al.* [5] describes a novel sensor for Lactate based on encapsulating an enzyme-phosphor within a permeable polymer. The sensor is based on measurement of fluorescence lifetime. The results presented give diffusion analysis to determine lactate transport through different polymer matrices with a view to optimising the sensor response.

Many thanks to the authors in this Special Issue for submitting papers which demonstrate the wide application and use of fluorescence-based technologies to different biosensing areas. Also much gratitude is owed to the reviewers of these articles for their diligence, thoughtful and constructive criticism.
